# Banana Ovate Family Protein MaOFP1 and MADS-Box Protein MuMADS1 Antagonistically Regulated Banana Fruit Ripening

**DOI:** 10.1371/journal.pone.0123870

**Published:** 2015-04-17

**Authors:** Juhua Liu, Jing Zhang, Wei Hu, Hongxia Miao, Jianbin Zhang, Caihong Jia, Zhuo Wang, Biyu Xu, Zhiqiang Jin

**Affiliations:** 1 Key Laboratory of Tropical Crop Biotechnology, Ministry of Agriculture, Institute of Tropical Bioscience and Biotechnology, Chinese Academy of Tropical Agricultural Sciences, Haikou, Hainan, China; 2 Haikou Experimental Station, Chinese Academy of Tropical Agricultural Sciences, Haikou, Hainan, China; Zhejiang University, CHINA

## Abstract

The ovate family protein named *MaOFP1* was identified in banana (*Musa acuminata* L.AAA) fruit by a yeast two-hybrid (Y2H) method using the banana MADS-box gene *MuMADS1* as bait and a 2 day postharvest (DPH) banana fruit cDNA library as prey. The interaction between MuMADS1 and MaOFP1 was further confirmed by Y2H and Bimolecular Fluorescence Complementation (BiFC) methods, which showed that the MuMADS1 K domain interacted with MaOFP1. Real-time quantitative PCR evaluation of *MuMADS1* and *MaOFP1* expression patterns in banana showed that they are highly expressed in 0 DPH fruit, but present in low levels in the stem, which suggests that simultaneous but different expression patterns exist for both *MuMADS1* and *MaOFP1* in different tissues and developing fruits. Meanwhile, *MuMADS1* and *MaOFP1* expression was highly stimulated and greatly suppressed, respectively, by exogenous ethylene. In contrast, *MaOFP1* expression was highly stimulated while *MuMADS1* was greatly suppressed by the ethylene competitor 1-methylcyclopropene (1-MCP). These results indicate that *MuMADS1* and *MaOFP1* are antagonistically regulated by ethylene and might play important roles in postharvest banana fruit ripening.

## Introduction

Transcription factors containing the MADS domain (for MCM1, AGAMOUS, DEFICIENS and SRF) are present in the majority of eukaryotic organisms [1]. Most plant MADS-box transcription factors are the MIKC-type, as they possess a modular structure where the MADS (M) domain is followed by an intervening (I), a keratin-like (K) and a C-terminal (C) domain [2–5]. Initially, MADS-box transcription factors were found to be major players in floral organ specification, but more recent studies revealed functions for MADS-box transcription factors in almost every developing process in plants, from root architecture formation [6], floral meristem determinacy and flowering [7], plant photosynthesis and nutrition metabolism [8], hormone signal transduction [9], to fruit development and ripening [10–17].

MADS-box transcription factors meditate their prominent roles in plant development through a complex network of protein-DNA and homo- or heterodimeric protein-protein interactions [18]. MADS-box transcription factors bind a specific target sequence, named the CArG box (CCA/T6GG) [4], in a promoter region. These transcription factors often bind as multimers, with each MADS-dimer binding one CArG box. Subsequently, the two dimers interact to form a tetramer and loop the DNA that lies between the two CArG boxes [19–21]. The first report on MADS-box transcription factor interactions was for TM6 and TM3 in tomato [22]. Researchers then focused on the interactions of MADS-box transcription factors in *Arabidopsis* and investigated the interaction map of more than one hundred *Arabidopsis* MADS-box transcription factors, which provided valuable information for identifying the functions of proteins involved in certain development processes [23–24]. Based on the high functional conservation of the MADS-box family members in many species and the fact that they show similar interaction patterns [25–27]. These complexes, however, do not seem to contain only MADS domain proteins. For instance, in *Arabidopsis*, molecular and genetic evidence suggests that AG-SEP complexes interact with the homeodomain transcription factor BELL1 (BEL1) to regulate ovule development, in particular to repress activity of the homeobox protein WUSCHEL in the ovule chalaza to control outer integument development [28]. Moreover, MADS-box proteins can interact with other non-MADS-box proteins such as SEUSS, Histone Fold Protein NF-YB and ubiquitin-activating (UBA) enzyme E1 protein MuUBA [29–31]. Despite the wealth of information concerning MADS-box protein interaction, there is little detailed knowledge of the mechanisms that are involved in the interactions of MADS-box proteins with other proteins to regulate plant growth and development.

Although yeast two-hybrid (Y2H) assays have been used in many plants such as *Arabidopsis*, tomato and *Petunia* to investigate protein-protein interactions, this technique has rarely been reported in banana [31]. In this study, an open reading frame of a D-class MADS-box gene named *MuMADS1* that was previously isolated from a banana suppression subtractive hybridization (SSH) library [32], was cloned into a bait vector (pGBKT7). *MuMADS1* expression is closely related to ethylene biosynthesis and fruit ripening and is mainly expressed in the carpel and during different ovary developing stages, while its expression in fruit is strongly induced by exogenous ethylene [32]. Using the MuMADS1 vector as bait in a Y2H screen, we identified an ovate family protein (OFP) gene named *MaOFP1*. The interaction mechanism of these two proteins in banana was further demonstrated by the Y2H method and Bimolecular Fluorescence Complementation (BiFC). The expression of *MuMADS1* and *MaOFP1* in different tissues and developing stages showed simultaneous but different patterns. Moreover, *MuMADS1* and *MaOFP1* expression in postharvest ripening stages following different treatments was antagonistically regulated at the transcriptional level by ethylene and 1-MCP. These results may increase the understanding of MADS-box gene mechanisms in regulating banana fruit development and ripening.

## Results

### Isolation of MaOFP1 by Y2H

**Fig 1:**
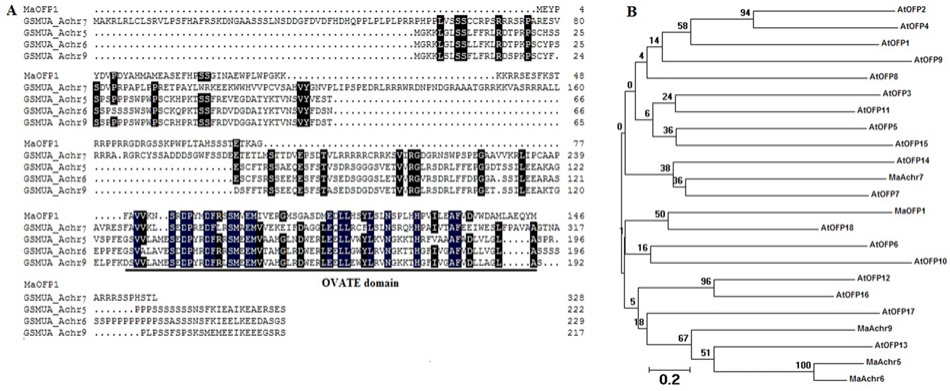
Sequence analysis of MaOFP1. (A) Sequence alignments of MaOFP1 with four other OFPs from the banana A genome. MaAchr7, 5, 6 and 9 represent OFPs located on chromosomes 7, 5, 6 and 9, respectively. Identical and similar amino acids are indicated by black and grey shading, respectively. The underlined sequence indicates the conserved ovate domain. (B) Phylogenetic analysis. The deduced amino acid sequences were aligned using Clustal W, and the dendrogram was drawn with the neighbor-joining method using MEGA software (Arizona State University, Tempe, AZ, USA). The number for each interior branch is the bootstrap percentage calculated from 1,000 replicates. The scale bar corresponds to 0.2 amino acid substitutions per residue. The protein sequences of the OFPs used for construction of the tree are listed in the GenBank database under the following locus or accession numbers: MaAchr7 (GSMUA_Achr5T17440_001), MaAchr5 (GSMUA_ Achr5 T17440_001), MaAchr6 (GSMUA_Achr6T07060_001), MaAchr9 (GSMUA_ Achr9T02650_ 001), AtOFP1 (*Arabidopsis thaliana*, NP_ 195804), AtOFP2 (NP_ 180599), AtOFP3 (NP_ 200644), AtOFP4 (NP_ 172174), AtOFP5 (NP_ 193618), AtOFP6 (NP_ 680125), AtOFP7 (NP_ 179440), AtOFP8 (NP_ 197466), AtOFP9 (NP_ 192312), AtOFP10 (NP_ 197616), AtOFP11 (NP_ 193222), AtOFP12 (NP_ 172033), AtOFP13 (NP_ 196102), AtOFP14 (NP_ 178114), AtOFP15 (NP_ 565833), AtOFP16 (NP_ 180770), AtOFP17 (NP_ 850144), AtOFP18 (NP_ 566967).

A Y2H assay was performed using *MuMADS1* as bait and 2 day postharvest (DPH) banana fruit cDNA library as prey. After identification on different minimal synthetic dropout (SD) supplements media, plasmids from the blue clones were isolated. Through Y2H we obtained a full-length ovate family protein gene designated *MaOFP1*, which contained a 542 bp sequence with a 438 bp full-length open reading frame (ORF) that encodes a protein of 146 amino acids. Bioinformatics analysis showed that MaOFP1 has a well-conserved ovate domain in its C-terminus and shared 37.91% identity with four other ovate family protein genes from the banana A genome that are located on chromosomes 7, 5, 6 and 9 (Fig 1A). Phylogenetic analysis showed that MaOFP1 was most closely linked with AtOFP18, and then AtOFP6 and AtOFP10 (Fig 1B). MaOFP1 showed a distant relationship with four other ovate family proteins from the banana A genome [33].

### Interaction of MuMADS1 and MaOFP1 *in vivo*

**Fig 2:**
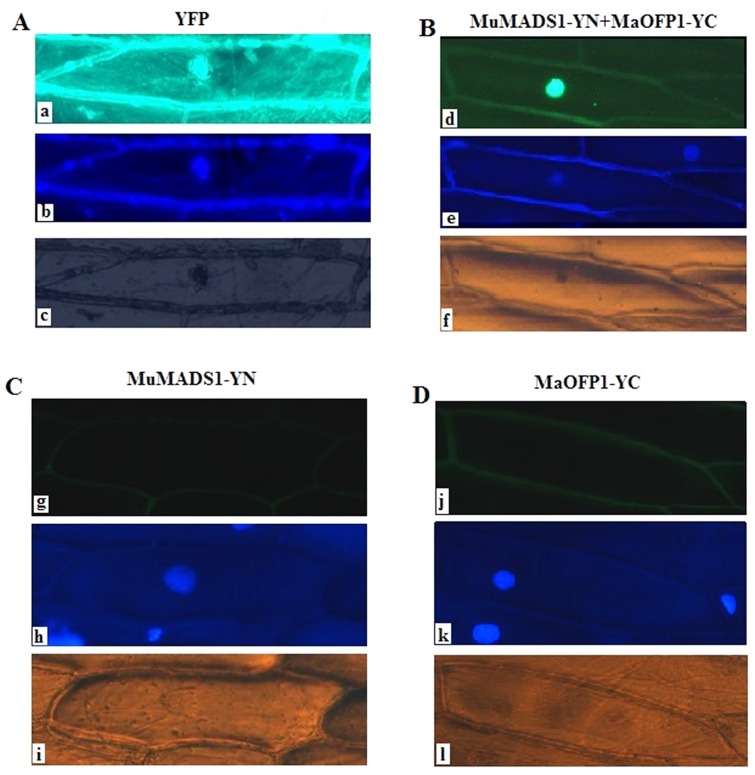
Interaction assay of MuMADS1 and MaOFP1 by the BiFC method. The free YFP protein (A) and *MuMADS1*-YN with *MaOFP1*-YC fusion proteins (B) were transiently expressed in onion epidermis cells and visualized with a ZEISS fluorescence microscope 24 h after bombardment with a gene gun. a and d: yellow fluorescence in cells, b and e: DAPI image, c and f: bright-field image. Panels C and D: Onion epidermis cells following bombardment with a gene gun as negative controls. C: *MuMADS1*-YN protein. D: *MaOFP1*-YC protein. g and j: yellow fluorescence. h and k: DAPI image. i and l: bright field image.

To analyze the interaction of MuMADS1 and MaOFP1 *in vivo*, *MuMADS1* and *MaOFP1* were fused in frame to the N-terminal and C-terminal fragments of YFP (YN and YC), respectively, under the control of the cauliflower mosaic virus 35S promoter (CaMV 35S). The two recombinant constructs were introduced into onion (*Allium cepa*) epidermal cells by the particle delivery method. Free YFP protein was distributed throughout the whole cell (nucleus and cytoplasm) (Fig 2A), whereas *MuMADS1*-YN with *MaOFP1*-YC accumulated mainly in the nucleus (Fig 2B). As negative controls, *MuMADS1*-YN and *MaOFP1*-YC were separately bombarded into onion epidermal cell, after which no fluorescence was detected (Fig 2C and 2D).

### The MuMADS1 K domain interacted with MaOFP1 in yeast

**Fig 3:**
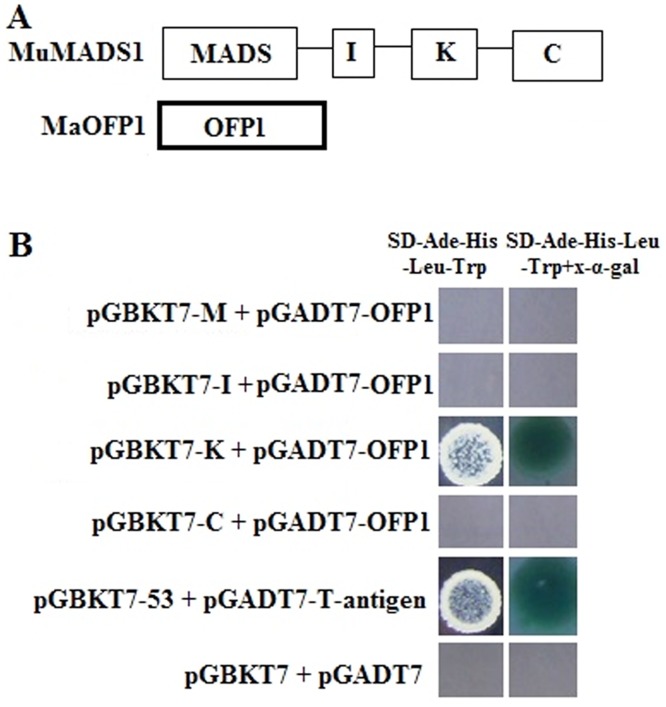
Y2H assays. (A) A schematic diagram illustrating the *MuMADS1* cDNA fragments encoding different portions of M, I, K and C that were fused to DNA sequences encoding the GAL4 DNA binding domain in the yeast vector pGBKT7. *MaOFP1* was cloned into pGADT7 as prey. (B) Positive interactions were determined through auxotrophic selection media SD/-Ade/-His-Leu/-Trp and SD/-Ade/-His-Leu/-Trp+ x-α-gal. The pGBKT7-53 vector used as a positive control interacted with the pGADT7-T-antigen while pGBKT7 empty vector as a negative control interacted with the pGADT7 empty vector.

To understand which domain of MuMADS1 interacted with MaOFP1, MuMADS1 was divided into four domains of M, I, K and C according to the sequence analysis. These four domains were each cloned into the pGBKT7 vector to fuse with the BD (bait domain). When the bait vectors containing the M, I, K and C domains and the prey vector containing *MaOFP1* were transformed into yeast strain AH109 and grown on auxotrophic selection media SD/-Ade/-His-Leu/-Trp+x-α-gal, blue clones appeared only for K+MaOFP1 and the positive control (pGBKT7-53+ pGADT7-T-antigen) as compared to the negative control. Other interactions of M + MaOFP1, I + MaOFP1 and C+ MaOFP1 yielded no blue clone growth. This result indicated that the K domain of MuMADS1 interacts with MaOFP1 in yeast (Fig 3A and 3B).

### Simultaneous but different expression of MuMADS1 and MaOFP1 in different tissues and fruit developmental stages

**Fig 4:**
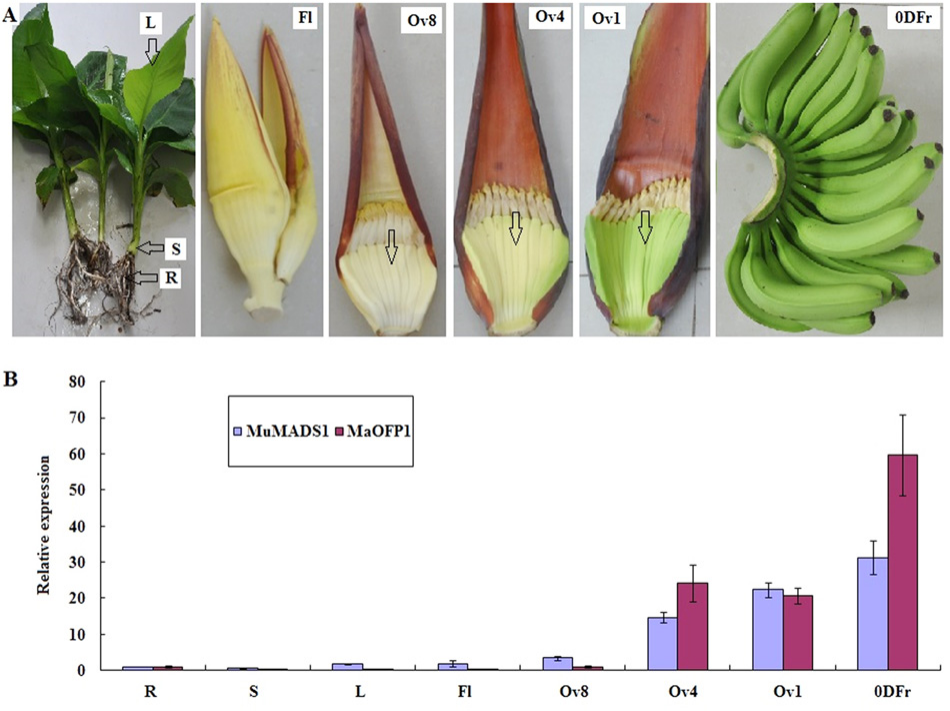
Relative expression of *MuMADS1* and *MaOFP1* in different tissues and developing fruits. (A) Different tissues and developing fruits: R: root; S; stem; L: leaf; Fl: flower; Ov8: Ovary 8 stage; Ov4: Ovary 4 stage; Ov1: Ovary 1 stage; 0DFr: 0 DPH fruits. (B)The x-axis represents the different tissues and developing fruits. The y-axis represents relative expression of *MuMADS1* and *MaOFP1* with respect to actin. Vertical bars on each column indicate SE from three replications.

RT-PCR analysis showed that both *MuMADS1* and *MaOFP1* were mainly expressed in the ovary 4 (Ov4), Ov1 stages and postharvest 0 DPH fruits. In 0 DPH fruits, *MuMADS1* and *MaOFP1* reached relative expression levels of 31.13 and 59.72, respectively. Both genes were poorly expressed in the stems with relative expression levels of 0.62 and 0.36, respectively (Fig 4). Together, these results suggest that *MuMADS1* and *MaOFP1* were simultaneous but different expression in different tissues and fruit developmental stages.

### Antagonistic regulation of MuMADS1 and MaOFP1 following ethylene and 1-MCP treatment

**Fig 5:**
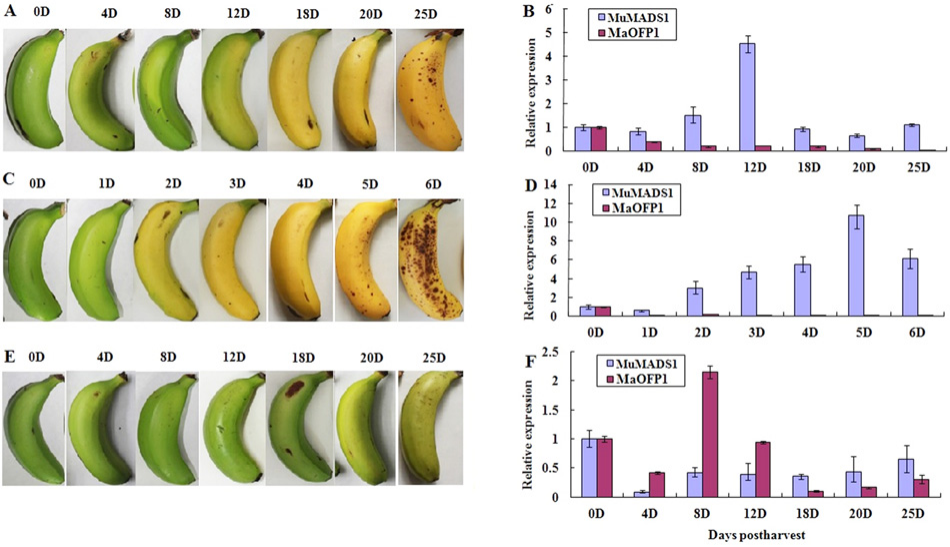
Relative expression of *MuMADS1* and *MaOFP1* in naturally ripened (A, B), ethylene-treated (C, D), and 1-MCP-treated (E, F) bananas. The x-axis represents the days postharvest and the y-axis represents relative expression of *MuMADS1* and *MaOFP1* with respect to actin. Vertical bars on each column indicate SE from three replications. When absent, the bars fall within the dimensions of the symbol.

The expression pattern of *MuMADS1* in naturally ripened fruits gradually increased and peaked at 4.5 at 12 DPH, after which the level decreased. However, *MaOFP1* expression gradually decreased with the fruit ripening process (Fig 5A and 5B). After treatment with exogenous ethylene, *MuMADS1* expression quickly increased and reached a peak of 10.69 at 5 DPH, which is 7 days earlier than naturally ripened fruits. However, the expression of *MaOFP1* was greatly suppressed and maintained at very low levels throughout the whole ripening process (Fig 5C and 5D). Meanwhile, expression of *MuMADS1* was strongly suppressed and maintained at low levels following treatment with the ethylene competitor 1-MCP, but *MaOFP1* expression was strongly increased and reached a peak of 2.1 at 8 DPH and then decreased (Fig 5E and 5F). This result indicated that *MuMADS1* and *MaOFP1* are antagonistically regulated by ethylene.

## Discussion

MADS-box transcription factors play crucial roles in plant developmental processes. MADS-box proteins can form homo- or heterodimeric protein-protein complexes to meditate their functions [18]. Moreover, a rice seed-specific NF-YB, which contains a histone fold motif, was identified as a partner of OsMADS18 by two-hybrid screening, which was the first report in plants that a MADS-box ternary complex protein formed from unrelated proteins [30]. Recently, Liu et al. [31] reported that banana MuMADS1, which is mainly expressed in developing ovaries and is closely related to ethylene biosynthesis and fruit ripening [32], could interact with another unrelated protein, MuUBA, and displayed co-expression patterns in different tissues, developing fruits and during the postharvest fruit ripening process. However, the mechanism for how MuMADS1 regulates banana fruit ripening remains unclear.

The OVATE gene is found exclusively in plants and encodes a protein with a putative nuclear localization signal and an approximately 70-aa C-terminal conserved domain, which was also known as the Domain of Unknown Function 623 (DUF623) [34]. Subsequently, this domain was designated as the OVATE domain, and proteins containing this domain were designated as Ovate Family Proteins (OFPs) [35]. The OVATE gene was first identified as an important regulator of fruit shape in tomato, in which a naturally occurring premature stop codon in OVATE results in pear-shaped fruit with longitudinal elongation and neck constriction [34]. Gene homologues containing the conserved OVATE domain have since been found in *Arabidopsis* (AtOFPs) and were shown to regulate plant growth and development [35–39].

AtOFPs were shown to have close functional interactions with three amino acid loop extension (TALE) homeodomain proteins, with AtOFP1 and AtOFP5 known to regulate the sub-cellular localization of TALE homeoproteins [35]. AtOFP1 has been reported to function as an active transcriptional repressor of AtGA20ox1 in the gibberellin (GA) biosynthesis pathway, where it suppresses cell elongation [37]. A recent study also indicated that AtOFP1 interacts with AtKu70, a protein involved in DNA repair through the non-homologous end-joining pathway [38]. Similar to AtOFP1, AtOFP4 acts as a transcriptional repressor and has been proposed to form a functional complex with KNAT7, one of four class II *Arabidopsis* KNOTTED1-like *Arabidopsis thaliana* (KNAT) members [40–41], to regulate secondary cell wall formation [39]. AtOFP5 was reported to interact with both BLH1 and KNAT3, which respectively belong to the BELL and KNOX subclasses of TALE homeodomain proteins [35], and can act as a regulator of the BELL–KNOX TALE complex that is involved in normal embryo sac development in *Arabidopsis* [36]. Recently, a genome-wide analysis of AtOFPs revealed that they have conserved functions as transcriptional repressors, with overexpression leading to a number of abnormal phenotypes, implying that they have novel roles in regulating plant growth and development [38]. In pepper (*Capsicum annuum*), a relative of tomato in the Solanaceae family, an OVATE family member (CaOvate) was also shown to be involved in determining fruit shape by negatively affecting the expression of CaGA20ox1 [42]. However, our knowledge concerning this protein relative to other OVATE family members in *Arabidopsis* and other plants remains relatively poor.

In this study, we identified an ovate family protein MaOFP1 by Y2H using banana MuMADS1 as bait and a banana 2 DPH cDNA library as prey. We then focused on the interaction mechanism of these two proteins and their expression patterns. MaOFP1 contains a conserved ovate domain, which might be necessary for its function and indicated that it is an OFP (Fig 1A). The close link between MaOFP1 and AtOFP18 suggested that they may have similar functions (Fig 1B). The distant evolution of MaOFP1 from four other OFPs in the banana A genome indicated that MaOFPs might have undergone a divergent expansion [43]. The interaction of MuMADS1 and MaOFP1 was further demonstrated by Y2H and BiFC, which represents one of the most advanced and powerful tools for studying and visualizing protein–protein interactions in living cells [44]. First, MuMADS1 was split into four regions of M, I, K and C. After reconstitution of these four regions and *MaOFP1* into bait and prey vectors, respectively, and subsequent transformation into yeast, the interaction of the K domain of MuMADS1 with MaOFP1 was again demonstrated by the appearance of blue clones upon cultivation on auxotrophic selection media SD/-Ade/-His-Leu/-Trp+ x-α-gal (Fig 3), which was consistent with the predicted result [4–5]. Using the BiFC method, *MuMADS1*-YN and *MaOFP1*-YC were co-bombarded into onion epidermal cells with a gene gun. YFP fluorescence was observed mainly in the nucleus, indicating that a direct physical interaction between MuMADS1 and MaOFP1 brought the split YFP fragments together in the nucleus. Using this method, we demonstrated that the MuMADS1 and MaOFP1 proteins localized to the nucleus, suggesting the possibility that MuMADS1 and MaOFP1 are transcription factors, which is consistent with a recent study on the cotton GhAGL15s MADS-box protein and tomato SLMBP21 MADS-box protein [45–46]. There are at least five MaOFPs in banana genome (Fig 1), and in this study, it was found that MuMADS1 could interact with MaOFP1 to regulate banana fruit ripening. However, whether MuMADS1 could interact with the other four MaOFPs requires further investigation.

The characteristics of these two proteins at the transcriptional level were further investigated. As shown in Fig 4, both *MuMADS1* and *MaOFP1* were highly expressed at the Ov4, Ov1 stages and in 0 DPH fruits, which is when the banana fruit quickly develops and ripens, but was expressed only at low levels in root and stem. This result was consistent with our previous report showing that *MuMADS1* is mainly expressed in developing ovaries and rarely or not expressed in roots, as indicated by semi-RT PCR results [32]. In addition, *MuMADS1* and *MaOFP1* were shown to display simultaneous but different expression in different tissues and developing fruits.

Banana is a typical climacteric fruit with ripening greatly stimulated by exogenous ethylene and suppressed by 1-MCP, an ethylene competitor that binds competitively to the ethylene receptor [47–48]. In naturally ripened banana fruits, endogenous ethylene slowly increases and then reaches a peak [49]. MuMADS1 is closely related to ethylene biosynthesis and fruit ripening [32]. In this study, the changes in *MuMADS1* expression paralleled ethylene biosynthesis and fruit ripening, while *MaOFP1* expression followed an opposite pattern (Fig 5B). Moreover, the expression level of *MuMADS1* was dramatically induced by exogenous ethylene and suppressed by 1-MCP. On the other hand, *MaOFP1* expression was greatly suppressed by exogenous ethylene and increased by 1-MCP (Fig 5D and 5F). This result strongly suggests that MuMADS1 antagonistically interacts with MaOFP1 to regulate banana fruit ripening. However, the way in which MuMADS1 is regulated by MaOFPs requires additional investigations using a plant transient assay system.

In conclusion, MuMADS1 interacted with MaOFP1 through the K domain. These two proteins were then subsequently localized to the nucleus where they act antagonistically to regulate banana fruit ripening.

## Materials and Methods

### Plant materials and treatments

Banana (*Musa acuminata* L. AAA group, cv. Brazilian) roots, stems, leaves, flowers and developing ovaries were obtained from the Institute of Tropical Bioscience and Biotechnology banana plantation (Chengmai, Hainan, 20N, 110E). Fruits were harvested at the mature green stage (100–110 d after flower shooting). Banana hands having a similar developmental stage were selected and five fingers from the hands were divided into three groups for different treatments. A control group was allowed to ripen naturally; the second and third groups were exposed for 12 h to 100 μL L^-1^ ethylene and 1 μL L^-1^ 1-MCP (Ethylblock, Rhom, and Haas, USA), respectively [47]. The treated materials were allowed to ripen at 25°C and subsequently frozen in liquid nitrogen and stored at -80°C before extraction of total RNA. Three biological replicates, with three flowers, ovaries, fruits, pieces of leaves or five grams of root and stem tissues for each replicate, were performed.

### Y2H screen

Y2H screening was performed using a MATCHMAKER GAL4 Two-Hybrid System 3 according to the manufacturer’s protocol (Clontech, http://www.clontech.com/). A fragment of *MuMADS1* was amplified by PCR with the primer pairs P1: 5′-CGGAATTCGATGGGAAGGGGTAAGAT-3′ and P2: 5′-CGGTCGA CTCTGATCCTTGTGATGC-3′. The PCR products were digested by *Eco*RI and *Sal*I and cloned into the *Eco*RI- *Sal*I site of the pGBKT7 bait vector and then immunodetected. In parallel, a 2 DPH cDNA expression library was constructed from 2 DPH banana fruit total RNA according to the manufacturer’s instructions (Clontech, http://www.clontech.com/). Subsequently, yeast cells containing the bait construct were transformed with the 2 DPH ‘‘prey” cDNA library according to the CLONTECH protocol. In total, 1.5×10^6^ individual yeast transformants were generated.

### Cloning and sequence analysis of MaOFP1

Single clones obtained by Y2H were identified again on different minimal media with SD/-Leu/-Trp, SD/-His/-Leu/-Trp, SD/-Ade/-His/-Leu/-Trp and SD/-Ade/-His/-Leu/-Trp+x-α-gal. An ovate family protein gene was amplified using long distance (LD) PCR primers P1: 5′-CTATTCGATGATGAAGATACC CCACCAAACCC-3′ and P2: 5′-GTGAACTTGCGGGGTTTTTCAGTATCTACG ATT-3′ and the Advantage 2 PCR Polymerase Mix (Clontech). The thermal cycling conditions were 94°C for 1 min followed by 30 cycles of 94°C for 30 s, and 68°C for 3 min. The amplified product was then inserted into the PMD-18T (Takara) vector, sequenced and the sequence analyzed by BLAST (http://ncbi.nlm.nih.gov/blast). Putative conserved domains were detected at NCBI (http://www.ncbi.nlm.nih.gov/Structure/cdd/wrpsb.cgi). Sequences from the banana A genome were aligned using DNAMAN software. The neighbor-joining method was used to construct the phylogenetic tree with MEGA software (Arizona State University, Tempe, AZ, USA).

### BiFC assay

A *MuMADS1* cDNA fragment was subcloned into the *Bam*HI/*Sal*I sites of pXY106 (kindly provided by Dr. Wang Xuelu, Fudan University; Shanghai, China) containing the N-terminal fragment of the yellow fluorescent protein (YFP, YN), and a *MaOFP1* cDNA fragment was subcloned into the *Kpn*I/*Xba*I sites of pXY104 (kindly provided by Dr. Wang Xuelu, Fudan University; Shanghai, China) containing the C-terminal fragment of the YFP (YC). The resulting two plasmids, pXY106-*MuMADS1* (*MuMADS1*-YN) and pXY104-*MaOFP1* (*MaOFP1*-YC), were co-bombarded with gold particles (Bio-Rad) into onion (*Allium cepa*) epidermal cell layers as described [50]. The CaMV35S-YFP plasmid was used as a control. After incubation at 25°C for 24 h in darkness, the epidermal cell layers were viewed with a Carl ZEISS (Axio Scope, Germany) fluorescence microscope. DNA staining was performed with an aliquot of culture medium containing 1 mg mL^–1^ 4', 6-diamido-2-phenylindole (DAPI).

### Analysis of MuMADS1 and MaOFP1 interacting domains in yeast

Four domains: M (50 aa), I (40 aa), K (50 aa) and C (95 aa) of *MuMADS1* were cloned into pGBKT7 to fuse with the bait domain (BD) using the primers (MP1: 5′-CGGAATTCATGGGAAGGGGTAAG-3′, MP2: 5′-CGGTCGACGCTGGAGA ACACA-3′; IP1: 5′-CGGAATTCCGCGGCAGGCTATATG-3′, IP2: 5′-CGGTCGA CGTAGTGCTGAGAATTG-3′; KP1: 5′-CGGAATTCTATCAGCAAGAATCTG C-3′, KP2: 5′-CGGTCGACTCTGATCCTTGTGATGC-3′, CP1: 5′-CGGAATT CTCGAAGAAGCATGAGC-3′, CP2: 5′-CGGTCGACCGCCTTGGTCTCATAGC C-3′). The PCR products were digested with *Eco*RI and *Sal*I and cloned into the *Eco*RI- *Sal*I site of the pGBKT7 bait vector. *MaOFP1* was cloned into pGADT7 to fuse with the activation domain (AD) of GAL4. Self-activation was assayed on selective synthetic dropout medium plates (SD/-Trp, SD/-Trp/-His, SD/-Trp/-His+X-α-gal). Then, combinations were simultaneously transformed into the yeast strain AH109 according to the protocol. The transformants containing plasmids pGADT7 and pGBKT7 were used as a negative control. The interactions were judged by growth on selective medium (SD/-Ade/-His/-Leu/-Trp+x-α-gal) according to the manufacturer’s protocol (Clontech, http://www.clontech.com/).

### RNA extraction and cDNA synthesis

Total RNA was extracted from the roots, stems, flowers, leaves, developing ovaries and fruits using a modified cetyltrimethylammonium bromide (CTAB) method [51]. First-strand cDNA was synthesized using the SMART PCR cDNA Synthesis Kit (Clontech, Palo Alto, CA, USA) according to the manufacturer’s instructions.

### Real-time RT-PCR

Total RNA was isolated from the pulp of banana fruit at different ripening stages following different treatments. Poly (A)^+^mRNA (200 ng) was converted into cDNA using the SMART PCR cDNA Synthesis Kit (Clontech) in a final volume of 20 μL, which subsequently served as the template for real-time PCR. Primer sets used in real-time RT-PCR for *MuMADS1* and *MaOFP1* were: MADS1RTP1: 5′-GTGGAGCTTCAGAGTGACAACATG-3′; MADS1RTP2: 5′-TGGTCCTGGTG ATGCGAGTAGTG-3′; OFP1RTP1: 5′-GCCATTAACGGCCCACAGTTC-3′; OFP1 RTP2: 5′-TGGTGGAGAGGTGAATTCAAG-3′. RT-PCR of actin was used as an internal control. For amplification of actin, the following primer set was designed: forward, 5'-TGTAGCAATTCAGGCTGTTCTT-3' and reverse, 5'-TCAGAGATGG CTGGAAGAGAAC-3'. SYBR Premix Ex Taq (TaKaRa) was used in 25 μL reactions with 0.5 μL ROX reference dye. Primers were used at 100 nM each with the equivalent of 100 ng reverse-transcribed RNA template per reaction. In all experiments, appropriate negative controls containing no template RNA were subjected to the same procedure to exclude or detect any possible contamination. Before proceeding with the actual experiments, a series of template dilutions was performed to determine the optimal template concentration to be used in the experiments for maximal target amplification.

Each quantitative real-time PCR was performed using the Stratagene Mx3000P (Stratagene, CA, USA) device and SYBR chemistry. The thermal cycling conditions were 94°C for 3 min followed by 40 cycles of 94°C for 7 s, 55°C for 10 s, and 72°C for 15 s. Reactions were performed in triplicate and data analyzed using MxProTM QPCR software (Stratagene, CA, USA). Actin was used as the calibrator or control sample to which *MuMADS1* and *MaOFP1* products were compared. Differences in quantification cycle (Cq) values between the *MuMADS1*, *MaOFP1* and *MaActin* transcripts were expressed as fold-change relative to actin.
